# “Left on read” examining social media users’ lurking behavior: an integration of anxiety and social media fatigue

**DOI:** 10.3389/fpsyg.2024.1406895

**Published:** 2024-08-02

**Authors:** Xiaoyu Liu, Ran Feng, Xiaobing Chen, Yu Yuan

**Affiliations:** ^1^School of Culture and Media, Shanxi College of Applied Science and Technology, Taiyuan, China; ^2^School of Humanities and Social Sciences, City University of Macau, Macao, China; ^3^School of Broadcast Announcing Arts, Communication University of Zhejiang, Hangzhou, China

**Keywords:** perceived overload, social comparison, privacy concern, social media fatigue, anxiety, lurking behavior

## Abstract

**Introduction:**

With the widespread use of social media, the behavior and mindset of users have been transformed, leading to a gradual increase in lurking users, which can impede the sustainable development of social media platforms. In this study, we aim to investigate the impact of intrinsic and extrinsic motivational factors on social media users’ anxiety, social media fatigue, and lurking behavior.

**Methodology:**

For the confirmation of these phenomena and to validate the theories, a structural equation model was constructed based on the SSO (Stressor-Strain-Outcome) theoretical framework. The model was then tested and validated with data from 836 valid online surveys. These data were analyzed using SPSS 27.0 and AMOS 24.0 software.

**Results:**

The results indicate that intrinsic motivations (such as social comparison and privacy concerns) and extrinsic motivations (including information overload, functional overload, and social overload) are positively associated with users’ lurking behavior through the mediating effects of social media fatigue and anxiety. Additionally, for the mediator variables, social media fatigue was found to be positively associated with anxiety.

**Discussion:**

These findings underscore the importance of social media platforms considering both intrinsic and extrinsic motivational factors to mitigate user anxiety and social media fatigue. By addressing these factors, platforms can foster user satisfaction and increase engagement, ultimately contributing to the sustainable development of social media platforms.

## 1 Introduction

Advances in Internet technology have fueled the rapid growth of social media platforms. WeChat, as one of the most popular social media platforms among Chinese users, seamlessly integrates various functions such as communication, payment, official accounts, Moments, Mini apps, and life services, fundamentally reshaping people’s lifestyles. Nevertheless, as social media becomes deeply intertwined with people’s professional, educational, and personal lives, users are increasingly fatigued by the constant demands for engagement such as liking, retweeting, commenting, bargaining, price cutting, voting, and other overwhelming social requests (Guo et al., 2020). Faced with the ever-expanding WeChat Moments and countless group chats, users are increasingly choosing to “leave on read” (a term commonly used in messaging and social media contexts to indicate that a person has read a message but has not responded to it), gradually transforming into a “silent presence” within the social media platform (Barnidge et al., 2023). These users receive information without actively responding, view information without providing feedback, read content without sharing it, and are often referred to as “lurkers” (Takahashi et al., 2003). Their behavior of exclusively searching or reading information that meets their needs on social networks, while seldom or never creating content, sharing information, or posting insights, is defined as lurking behavior (Edelmann, 2013). In the realm of social media, consistent user engagement and active content creation are essential to platform activity, and the presence of lurkers significantly hampers the platform’s sustainable development (Kang and Shin, 2008; Ortiz et al., 2018). Hence, identifying the underlying reasons for lurking is imperative for the advancement of social media platforms.

Existing literature attributes lurking behavior to factors such as computer anxiety (Osatuyi, 2015), technology overload (Karr-Wisniewski and Lu, 2010), information security awareness (Ortiz et al., 2018), and knowledge-collecting behavior (Nguyen et al., 2022). However, the majority of these studies concentrate on investigating individual factors, thereby neglecting the broader psychological motivations driving lurkers and the comprehensive impact of the external environment on their behavior. Thus, this study draws upon self-determination theory (Deci et al., 1991; Hung et al., 2011) to propose that user lurking behavior is influenced by a combination of intrinsic and extrinsic motivations.

This study aims to investigate the factors influencing WeChat users’ lurking behavior by addressing the following questions: First, what intrinsic and extrinsic motivations contribute to social media users’ lurking behavior amidst the prevalence of social media? Second, how do these motivating factors impact users’ lurking behavior and what tailored preventive measures can be implemented? To delve deeper into these inquiries, the study adopts the SSO model to uncover the underlying motivations driving social media users’ lurking behavior and to shed light on the essence of “left on read” behavior. By doing so, the research seeks to fill the gaps in existing literature concerning the complexities of social media users’ lurking behavior. Additionally, it strives to unveil users’ interaction patterns and communication needs within the virtual realm of social media, with the ultimate goal of optimizing the functionality of social media platforms and offering enhanced solutions to mitigate issues related to users’ lurking behavior. Through this investigation, it also aims to provide concrete recommendations for refining the functions of social media platforms and enhancing overall user experience in the virtual space.

## 2 Literature review

### 2.1 SSO model

The SSO model consists of stressor, strain, and outcome (Koeske and Koeske, 1993), with the strain mediating between the stressor and the behavioral outcome. This model delves into the micro-psychological processes within the stimulus-organism-response paradigm and investigates the impacts of various stressors on an individual’s psychology and behavior. Stressors in this context refer to environmental stimuli that induce stress in individuals, while strain manifests as negative disruptions in attention, cognition, and emotion that individuals experience when under the influence of a stressor. Outcomes signify the enduring feelings of stress or tension that individuals undergo when exposed to a stressor, resulting in sustained negative behavioral or psychological reactions (Hu et al., 2024).

The SSO model, serving as a comprehensive framework for explaining an individual’s intrinsic psychological perceptions and behavioral responses to environmental stimuli, is extensively utilized in the realms of psychosocial wellbeing (Dhir et al., 2018), emotional dissonance (Takahashi et al., 2003), social media fatigue (Dhir et al., 2019), and other health communication fields. In this study, intrinsic user stress and extrinsic social media stress collectively form a stressor for social media users, triggering a negative focal strain response that ultimately manifests as lurking behavior. Consequently, the utilization of the SSO model to explore the lurking behavior of social media users in this study is considered fitting.

### 2.2 The self-determination theory

The Self-Determination Theory (SDT) is a comprehensive theory of individual motivation, emotions, and personality qualities that focuses on the underlying reasons for individual behavior (Deci and Ryan, 2000). According to the theory, motivation serves as the driving force behind human behavior, determining the specific actions individuals take in response to external circumstances and internal needs. Previous research has demonstrated the significant impact of motivation on technology acceptance behavior (Teo et al., 1999; Venkatesh et al., 2002), job stress and employees’ well-being (Luo, 1999), as well as physical activity (Teo et al., 1999; Vallerand, 2001).

Individual motivation can be classified into three main types based on the perceived level of autonomy: intrinsic motivation, extrinsic motivation, and amotivation (Deci and Ryan, 2000). Intrinsic motivation stems from internal factors that bring satisfaction to an individual during a specific activity, reflecting an internal drive. On the other hand, extrinsic motivation is driven by external factors such as rewards, promotions, punishments, and coercion. Both intrinsic and extrinsic motivation play a crucial role in shaping an individual’s intentions and actions toward an activity (Olatokun and Nwafor, 2012). Therefore, this study examines both intrinsic and extrinsic motivations and their impact on lurking behavior in a holistic approach.

### 2.3 Social media lurking

As users become more knowledgeable and experienced in using social media, their engagement and contribution to social media platforms evolve over time (Hong et al., 2023). Online knowledge communities categorize participants into two groups: lurkers and posters. Lurkers are individuals who read posts without actively contributing, while posters are those who share knowledge within online communities (Nguyen, 2020, 2021). Some previous studies have portrayed lurking behavior as a negative behavior among social media users, characterized by a typical “free-riding” phenomenon (Sun et al., 2014), where lurkers consume information without actively sharing it, resulting in minimal contribution to the online community (Van Mierlo, 2014).

However, some scholars argue that lurking behavior serves as a social media participation strategy for users aiming to safeguard the privacy of their personal information within social media networks (Osatuyi, 2015). Permission to lurk is seen as an essential requirement for users to integrate into unfamiliar online communities, allowing them to understand and adapt to the social environment through passive observation upon initial entry (Neelen and Fetter, 2010). Building on scholars’ definitions of lurking behavior, this study defines lurking behavior as the observation-only behavior of social media users without active participation on social media platforms (Ortiz et al., 2018).

Various triggers prompt lurking behavior, some users perceive lurking as a method to protect their private information (Osatuyi, 2015; Ortiz et al., 2018), while others, concerned about their reputation within the community, opt to lurk to avoid comparisons with other users regarding information dissemination and knowledge contribution (Nisar et al., 2019). Moreover, when users experience information and social overload on social media, they may develop negative emotions like anxiety and fatigue, leading to a deliberate reduction in content contribution and engagement in information avoidance behaviors (Guo et al., 2020; Nguyen, 2020). Social media fatigue is also considered a trigger for lurking behaviors (Hong et al., 2023). Social media fatigue refers to the feelings of weariness, monotony, and ennui experienced by users during social media usage, influenced by personal, platform, and social factors (Maier et al., 2015).

However, the majority of existing studies have examined the reasons behind social media users’ lurking behavior from a singular standpoint, either negative or positive, thereby hindering a comprehensive understanding of the underlying factors contributing to such behavior. In an effort to address this gap, this study leverages the Self-Determination Theory (SDT) to investigate the impact of anxiety and social media fatigue as mediating variables on users’ lurking behaviors, taking into account two primary factors: intrinsic motivation and extrinsic motivation.

## 3 Research hypothesis and model

Motivation plays a pivotal role in influencing user behavior (Deci and Ryan, 2000). In this study, intrinsic and extrinsic motivations are closely examined. Intrinsic motivation, which stems from within an individual, is scrutinized through the lenses of social comparison and privacy concerns; whereas extrinsic motivation, originating from external sources, is analyzed in terms of information overload, functional overload, and social overload. These motivations are chosen for their ability to capture the essence of social media characteristics and user behaviors.

Social comparison unveils the negative emotional responses users experience when comparing themselves to more specialized individuals, potentially diminishing their positive engagement and resulting in lurking behavior (Nisar et al., 2019; Verduyn et al., 2020; Okano and Nomura, 2023). Privacy concerns signify users’ apprehensions regarding data protection. If users feel their personal data is being mishandled, they perceive privacy risks and are hesitant to engage in information sharing activities (Osatuyi, 2015; Ortiz et al., 2018).

Regarding extrinsic motivations, information overload manifests as users’ unease with the abundance of information on social media, potentially diminishing the perceived value of knowledge. Functional overload refers to the disruption in user behavior due to platform over functionality. Social overload amplifies the online burdens on users through excessive social demands, consuming time and attention, consequently resulting in lurking behavior (Guo et al., 2020; Liu et al., 2020; Hong et al., 2023).

Moreover, the inclusion of anxiety and social media fatigue as psychological strain responses more accurately captures the negative emotions contributing to users’ lurking behavior (Dhir et al., 2018; Hong et al., 2023; Li et al., 2024). Users experiencing anxiety due to factors like lack of expertise and self-confidence may refrain from engaging in sharing activities (Okano and Nomura, 2023). Social media fatigue symbolizes users’ weariness and burnout from prolonged social media use and the inability to find gratification in sharing activities, thus resulting in lurking behavior (Hong et al., 2023).

### 3.1 Intrinsic motivation

#### 3.1.1 Social comparison

Social comparison involves using others as a reference point to gage our own behavior in relation to theirs (ability comparisons) or to determine how we should think, feel, and act (opinion comparisons). These comparisons offer insights into our own abilities, social standing, and performance, as well as those of others (Festinger, 1954). In online social settings, users frequently engage in comparisons with other community members, evoking a range of emotions. Individuals typically focus on self-evaluation as the central aspect of social comparison (Lim and Yang, 2019). As users of social media platforms, individuals tend to concentrate on their impact within the community and may consciously or subconsciously measure themselves against those who have achieved greater success, leading to feelings of inadequacy and negative sentiment (Nisar et al., 2019). This behavior of social comparison diminishes self-confidence in one’s knowledge, abilities, and other facets of life, heightens anxiety, and diminishes motivation to participate in activities, potentially resulting in escalating fatigue and burnout associated with social media usage.

Moreover, upward social comparison (where individuals compare themselves to those in more favorable circumstances) represents a common social phenomenon (Okano and Nomura, 2023). On social media platforms, individuals engage in self-comparisons by observing the content (e.g., posts, photos, etc.) presented by others online, with the number of likes and comments serving as indications of individual social competence and popularity. This uptick in social comparison behavior notably intensifies the frequency of comparisons made by social media users, perpetuating feelings of social fatigue stemming from continual self-evaluation against others. Consequently, users’ interest in social interactions may dwindle, making lurking behavior more prevalent. Therefore, the following hypotheses are proposed:

*H*1a: Social comparison is positively associated with anxiety.

*H*1b: Social comparison is positively associated with social media fatigue.

#### 3.1.2 Privacy concerns

In today’s digital landscape, an increasing number of users are persistently mindful of the significance of online privacy issues, indicating their apprehensions about potential data breaches associated with social media use (Zhang et al., 2022). Despite this heightened awareness, numerous personal data points are susceptible to unauthorized exposure on social media platforms without users’ explicit consent, leading to heightened levels of privacy-related anxiety. Individuals troubled by privacy concerns often harbor suspicions that the online community and its members could potentially exploit their personal information, fostering a substantial sense of privacy risk that dissuades them from freely sharing information within the community (Kim et al., 2021). In a notable instance, Winder (2021) reported a data breach in which the personal data of 419 million Facebook users was compromised and exposed to unsecured databases in 2019. Additionally, many interface interactions necessitate users to click the “consent” button to proceed, inadvertently granting platforms permission to gather information about their browsing habits, potentially leading to unauthorized dissemination of data without the user’s explicit consent. This heightened awareness regarding privacy issues can contribute to psychological fatigue (Fan et al., 2020). Furthermore, privacy concerns may cultivate user distrust in social media usage. A study by Ortiz et al. (2018) explored the connection between personal privacy concerns and users’ behavioral intentions, noting that such concerns are likely to induce anxiety, subsequently influencing users to adopt negative usage patterns that ultimately culminate in reduced social media engagement. Therefore, the following hypotheses are proposed:

*H*2a: Privacy concerns are positively associated with anxiety.

*H*2b: Privacy concerns are positively associated with social media fatigue.

### 3.2 Extrinsic motivation

Building upon the technology overload framework proposed by Karr-Wisniewski and Lu (2010), Zhang et al. (2016) introduced three types of perceptual overload within social network service environments: system functional overload, information overload, and social overload. Perceptual overload has been highlighted as a significant precursor to various negative outcomes such as anxiety, exhaustion, regret, and fatigue (Guo et al., 2020; Huang et al., 2022; Li et al., 2022). This overload can instigate feelings of fatigue, subsequently influencing users’ intentions or behaviors toward discontinuing usage. Nevertheless, the impact of perceived overload on users’ lurking behavior, whether indirect or direct, remains ambiguous. Therefore, this study examines it as an extrinsic environmental stimulus that motivates lurking behavior.

#### 3.2.1 Functional overload

While the current features of social media platforms generally cater to the basic requirements of users, platforms frequently introduce new features via system updates to address issues, improve the interface, or enhance overall user experience. As the functionality of these platforms continues to expand, there is a risk of surpassing the genuine needs of users, leading to functional overload (Thompson et al., 2005; Zhou and Xie, 2023). Platform operators often introduce these new features with the intention of enhancing the user experience; however, some features may not align with users’ actual needs, potentially adding to users’ burdens and anxieties (Fu et al., 2020). Given that many users engage with multiple social media platforms simultaneously, they must invest considerable time and effort in familiarizing themselves with these new features that may exceed their requirements. If the benefits derived from learning and utilizing these additional features do not outweigh the associated costs, users may experience feelings of monotony and fatigue (Lee et al., 2016). Furthermore, certain added features could heighten the complexity of social media platforms, diminishing usability and, consequently, fostering user dissatisfaction. Therefore, the following hypotheses are proposed:

*H*3a: Functional overload is positively associated with anxiety.

*H*3b: Functional overload is positively associated with social media fatigue.

#### 3.2.2 Information overload

In the era of rapid technological advancement, users are inundated with vast quantities of intricate and often indistinguishable information across social media platforms. While information serves to alleviate uncertainty, an excessive influx of data can exacerbate uncertainty and impose cognitive burdens (Hogan and Brashers, 2015; Zhang et al., 2016). Information overload ensues when users are inundated with information that surpasses their limited processing capabilities, eliciting negative emotional responses (Lee et al., 2016). This overload stands as a primary driver of user fatigue; heightened perceptions of information overload correspond to increased stress levels and subsequent fatigue (Kim and Park, 2015). Users may experience discontent stemming from a perceived lack of effective information filtering mechanisms on the platform. Moreover, when information surpasses users’ cognitive thresholds, an excess of irrelevant and nebulous data compounds user fatigue, potentially leading to mental distress, stress, and anxiety (Fu et al., 2020). Therefore, the following hypotheses are proposed:

*H*4a: Information overload is positively associated with anxiety.

*H*4b: Information overload is positively associated with social media fatigue.

#### 3.2.3 Social overload

The advent of mobile technology has facilitated pervasive social interaction and communication through social networks. Research by Dunbar (1992) suggests that individuals can maintain stable relationships with up to 150 friends. However, the prevalence of social media in everyday life has led many users to accumulate online connections far surpassing Dunbar (1992) proposed limit. Within the realm of social media, users are often inundated with various social demands from their network, and social overload manifests when individuals are confronted with more requests than they can manage (Zhang et al., 2016). Social overload can disrupt users’ regular activities, divert their attention, induce feelings of stress and anxiety, impede their engagement, and foster unsustainable patterns of social media use (Cao and Sun, 2018). Consequently, users may find themselves struggling to cope with the demands placed upon them, leading to heightened levels of anxiety and fatigue. Therefore, the following hypotheses are proposed:

*H*5a: Social overload is positively associated with anxiety.

*H*5b: Social overload is positively associated with social media fatigue.

### 3.3 Anxiety

Anxiety is a prevalent emotional state that arises when individuals find themselves in uncertain circumstances, experiencing internal tension and distress, while the autonomic nervous system initiates coping mechanisms in response to perceived threats (Bekker et al., 2003). In the realm of social media, anxiety stands out as a common negative psychological repercussion often stemming from social media overload (Dhir et al., 2018). This state of anxiety can prompt various perceptual and evaluative hindrances, such as distortions in perception, hyper-vigilance toward potential dangers and threats, as well as impairments in rational judgment and information processing abilities. Within the sphere of social media, anxiety may deter users from actively engaging in information sharing and interactive pursuits, resulting in adverse usage behaviors (Huang et al., 2022). Moreover, individuals may harbor concerns about privacy breaches or subpar content quality when posting on social media, impacting the construction of their personal image. Consequently, anxious users may gravitate toward an observer role or ‘lurking’ mode (Okano and Nomura, 2023) to mitigate the risk of errors and bolster their self-efficacy on social media platforms (Li et al., 2024). Consequently, users grappling with anxiety on social media are prone to abstain from active participation in discussions or knowledge dissemination, opting instead to maintain a low profile. Therefore, the following hypothesis is proposed:

*H*6: Anxiety is positively associated with lurking behavior.

### 3.4 Social media fatigue

Social media fatigue manifests when users grow disenchanted with the myriad features and overwhelming content present on social media platforms, prompting them to seek respite (Guo et al., 2020). This psychological weariness, known as social media fatigue, is intrinsically linked to the emergence of anxiety. Previous studies have highlighted that individuals may experience cognitive impairment when grappling with burnout, hindering their ability to effectively regulate their emotions (Lee et al., 2016). The adverse consequences of social media fatigue may drive users toward a passive role on social media platforms, exacerbating both psychological and physical exhaustion, potentially prompting them to temporarily or permanently retreat from online interactions to alleviate distress (Fan et al., 2020; Hsu et al., 2023). Scholars also posit that fatigue could elicit users’ discontent with social media, serving as a key catalyst for negative behaviors like discontinuing usage (Zhang et al., 2016, 2022). Therefore, the following hypotheses are proposed:

*H*7a: Social media fatigue is positively associated with anxiety.

*H*7b: Social media fatigue is positively associated with lurking behavior.

**Figure 1:**
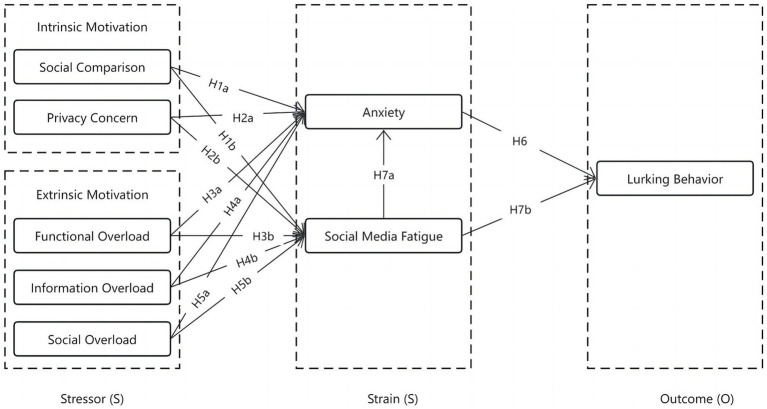
Conceptual framework and research hypotheses.

In summary, utilizing the SSO model and the preceding discussion, this study puts forth a theoretical model for examining the lurking behavior of social media users. The conceptual framework is visually depicted in Figure 1.

## 4 Methodology

### 4.1 Measurement development

A two-part questionnaire was meticulously crafted to gather data from the participants. The first segment aimed to capture demographic information including gender, age, education level, daily frequency and duration of WeChat usage, WeChat experience, and the number of WeChat contacts. The subsequent part encompassed all the constructs outlined in the research model. The research inquiries in this study were adapted from established scales, with slight modifications made to align them with the research context and the practical experiences of WeChat users.

The questionnaire underwent a rigorous validation process, inclusive of back-translation and pre-testing procedures. Initially, the English questionnaire was translated into Chinese and subsequently retranslated back into English by an independent individual to refine and ensure the content’s accuracy. A panel of experts carefully scrutinized the data collection tool to evaluate the appropriateness of the chosen indicators for representing the constructs. Following this, the questionnaire underwent a pre-test involving 60 WeChat users to validate the efficacy of the tool. Based on the feedback from the pre-survey, suggestions from respondents and professional scholars were incorporated and ambiguous statements in the questions were corrected to ensure the accuracy and clarity of the questionnaire. The final measurement items are shown in Supplementary Table 1.

### 4.2 Sample and data collection

We selected WeChat users in China for our study due to the platform’s widespread popularity (Chen et al., 2019). The survey took place in January and February of 2024. Random samples were obtained through the online survey platform, Credamo, which is known for its reliability and wide usage in China. Respondents who completed valid questionnaires were offered material or monetary incentives. A total of 862 responses were collected. First, questionnaires completed in under 3 min were eliminated to ensure thoughtful responses. Second, all responses were carefully scrutinized, and those indicating “I do not use WeChat” in the first question were excluded. Third, 14 responses were disqualified due to missing data. Ultimately, 836 completed surveys were obtained, resulting in a recovery rate of 96.98%.

### 4.3 Measurement of variables

The question items in this study were sourced from established scales, with minor adjustments made to tailor the inquiries to the specific research context. All items were assessed using a 5-point Likert scale ranging from strongly disagree (1) to strongly agree (5). Among these, social comparison, privacy concern, information overload, functional overload, and social overload were considered independent variables, while social media fatigue and anxiety served as mediating variables, and lurking behavior was the dependent variable. The detailed measurements are outlined below:

The measurement scales for social comparison were adapted from studies by Nisar et al. (2019) and Jabeen et al. (2023).The measurement scale for privacy concerns was sourced from the work of Cain and Imre (2022).

Measurement scales for functional overload were adopted from Guo et al. (2020) an Lee et al. (2016); information overload measurements were derived from Zhang et al. (2016) and Guo et al. (2020); social overload scales were based on studies by Guo et al. (2020) and Fu et al. (2020).

The measure of anxiety was taken from the research of Liu et al. (2020). Social media fatigue measurements were sourced from studies by Jabeen et al. (2023) and Kaur et al. (2021).

The scale for lurking behavior was adapted from studies conducted by Zhang et al. (2021) and Hong et al. (2023). The final measurement items can be found in Supplementary Table 1.

Moreover, individual-level differences, particularly sociodemographic variables such as age (Reer et al., 2019), may influence behaviors and outcomes related to social media use. Hence, in line with prior research (Dhir et al., 2021), we included age, gender, and education level as control variables.

### 4.4 Demographic data

The demographics of the sample are outlined in Table 1 below. The data reveals that there were 388 males (46.4%) and 448 females (53.6%). Regarding age distribution, individuals aged 21 to 30 accounted for 36.1%, closely followed by the 31 to 40 age group at 34.4%. This distribution aligns with the typical age demographics of social media users, predominantly consisting of middle-aged and young individuals. Furthermore, a significant portion of the sample indicated high frequency and duration of WeChat usage, with 60.2% using WeChat more than 10 times a day, 63% using the platform for over 2 h daily, 60.2% having over 5 years of WeChat experience, and 82.1% having more than 100 WeChat friends.

**Table 1:** Demographic characteristics of participants (*N* = 836).

Characteristics	Frequency	Percent (%)
**Gender**		
Male	388	46.4
Female	448	53.6
**Age**		
21–30	302	36.1
31–40	288	34.4
41–50	104	12.4
51–60	123	14.7
Over 60	19	2.3
**Education level**		
Elementary school and below	19	2.3
Junior high school	31	3.7
General high school/secondary school/technical school/vocational high school	77	9.2
Specialized education	235	28.1
Undergraduate degree	349	41.7
Master degree	98	11.7
Doctor degree	27	3.2
**Frequency of using WeChat**		
Under 2 times a day	9	1.1
2–4 times a day	42	5.0
5–7 times a day	112	13.4
8–10 times a day	170	20.3
Over 10 times a day	503	60.2
**Daily time spent on WeChat**		
Less than 2 h	309	37.0
2 h-4 h	374	44.7
4 h-6 h	105	12.6
Over 6 h	48	5.7
**Experience with using WeChat**		
Under 2 years	16	1.9
2–3 years	35	4.2
3–4 years	80	9.6
4–5 years	202	24.2
Over 5 years	503	60.2
**Number of WeChat friends**		
Less than 50	40	4.8
50–100	109	13.0
100–150	104	12.4
150–200	190	22.7
More than 200	393	47.0

## 5 Results

For this study, data analysis was conducted using SPSS 27.0 and AMOS 24.0 software. Initially, the acquired data underwent descriptive statistical analysis, reliability and validity tests. Subsequently, structural equation modeling was utilized for validation factor analysis, fit analysis, and path coefficient analysis of the model. Finally, the study examined the mediating role of social media fatigue and anxiety in a chain mediation analysis.

### 5.1 Validation factor analysis

Before proceeding with the structural equation modeling, aside from evaluating the questionnaire’s reliability, assessing the scale’s validity is essential, encompassing content validity, convergent validity, and discriminant validity. Firstly, considering that the scales used to measure latent variables in this study are sourced from established and well-tested scales, the content validity is deemed satisfactory. Secondly, examination of Table 2 reveals that all factor loadings exceed 0.6, with a KMO coefficient of 0.940, and an overall Bartlett’s ball test chi-square value of 20278.000 (df = 861, *p* < 0.001), indicating a cumulative variance contribution ratio of 66.121%. Moreover, the combined reliability (CR) of all latent variables surpassed 0.8, while the average variance extracted value (AVE) was consistently above 0.5, signifying strong internal consistency and aggregation within the questionnaire. Additionally, as illustrated in Table 3, to assess the differentiation between latent variables, following the approach suggested by Fornell and Larcker (1981), comparing correlation coefficients between the square root of AVE and the corresponding latent variables is necessary to test discriminant validity. The analysis reveals that the square root of AVE for each latent variable exceeds the respective correlation coefficient, affirming the scale’s clear differentiation validity.

**Table 2:** Reliability and convergent validity.

Variable	Item	Factor loading	Cronbach’s α	CR	AVE
Social comparison (SC)	SC1	0.903	0.899	0.901	0.567
	SC2	0.726			
	SC3	0.726			
	SC4	0.737			
	SC5	0.704			
	SC6	0.72			
	SC7	0.737			
Privacy concern (PC)	PC1	0.912	0.888	0.891	0.578
	PC2	0.72			
	PC3	0.738			
	PC4	0.73			
	PC5	0.715			
	PC6	0.73			
Functional overload (FO)	FO1	0.891	0.879	0.883	0.602
	FO2	0.741			
	FO3	0.751			
	FO4	0.75			
	FO5	0.738			
Information overload (IO)	IO1	0.898	0.870	0.874	0.584
	IO2	0.727			
	IO3	0.703			
	IO4	0.718			
	IO5	0.761			
Social overload (SO)	SO1	0.92	0.844	0.853	0.596
	SO2	0.715			
	SO3	0.733			
	SO4	0.701			
Anxiety (AM)	AM1	0.89	0.839	0.847	0.583
	AM2	0.714			
	AM3	0.72			
	AM4	0.716			
Social media fatigue (SMF)	SMF1	0.913	0.893	0.895	0.59
	SMF2	0.724			
	SMF3	0.732			
	SMF4	0.738			
	SMF5	0.708			
	SMF6	0.777			
Lurking behavior (LUK)	LUK1	0.87	0.872	0.876	0.586
	LUK2	0.749			
	LUK3	0.727			
	LUK4	0.758			
	LUK5	0.716			

*N* = 836. CR, Composite reliability. AVE, Average Variance extracted.

**Table 3:** Discriminant validity of constructs.

	M	SD	LUK	SMF	AM	SO	FO	IO	PC	SC
LUK	2.602	0.896	0.766							
SMF	2.678	0.925	0.58**	0.768						
AM	2.627	0.921	0.518**	0.397**	0.764					
SO	2.586	0.947	0.501**	0.461**	0.394**	0.772				
FO	2.64	0.964	0.573**	0.433**	0.424**	0.412**	0.776			
IO	2.705	0.941	0.545**	0.385**	0.421**	0.378**	0.457**	0.764		
PC	2.608	0.918	0.498**	0.433**	0.411**	0.38**	0.397**	0.436**	0.76	
SC	2.638	0.897	0.501**	0.377**	0.38**	0.33**	0.393**	0.363**	0.4**	0.753

M, Mean; SD, Standard Deviation. SC, Social comparison; PC, Privacy concern; FO, Functional overload; IO, Information overload; SO, Social overload; AM, Anxiety; SMF, Social media fatigue; LUK, Lurking behavior.*p < 0.05, **p < 0.01.Numbers on the diagonal are the square roots of the latent variable Average Variance Extracted (AVE); numbers below the diagonal are the correlation coefficients between latent variables.

The discriminant validity test results are presented in Table 3 below. The diagonal line represents the mean variance extraction (AVE) values, which are higher than the correlation coefficients on the non-diagonal line. This suggests that the scale demonstrates good discriminant validity, and the data provides support for further testing and analysis.

Table 3 also demonstrates a significant correlation (*p* < 0.01) between social comparison, privacy concerns, information overload, functional overload, social overload, anxiety, social media fatigue, and lurking behaviors. This indicates that the latent variables exhibit both discriminative and correlative relationships with each other, thereby suggesting that the data’s discriminative validity of the scale is optimal.

### 5.2 Common method bias

In this study, common method bias was mitigated through procedural measures, such as ensuring anonymity in measurements. Data obtained were evaluated for common method bias using the Harman single-factor test. The unrotated exploratory factor analysis results revealed a total of eight factors, each with eigenvalues >1. The highest proportion of variance explained by a factor was 32.87%, which falls below the 40% threshold. Thus, it can be inferred that the results of this study were not significantly influenced by common method bias.

### 5.3 Model fitting and hypothesis testing

The purpose of this study is to discuss the mechanisms of intrinsic motivation based on social media users (WeChat) and extrinsic motivation for social media perceptual overload on users’ lurking behavior, and to test the mediating role of anxiety and social media fatigue from a social psychology perspective. To achieve this, AMOS 24.0 was utilized to construct a structural equation model (SEM) in order to analyze the relationships between latent variables. Initial scrutiny involved assessing and refining the model fit based on the Modification Index (MI) values provided by the structural equations. As indicated in Table 4, the chi-square degrees of freedom ratio falls below the critical value of 3, and key fit indices such as GFI, CFI, NFI, IFI, and TLI surpass the threshold of 0.9. Additionally, the RMSEA value is below 0.08, collectively suggesting a strong fit for the proposed model.

**Table 4:** Model fit indices (*N* = 836).

Fit indices	x^2^/df	GFI	CFI	NFI	IFI	TLI	RMSEA
The threshold value	<3	>0.9	>0.9	>0.9	>0.9	>0.9	<0.08
The actual value	1.938	0.920	0.963	0.926	0.963	0.959	0.034

**Figure 2:**
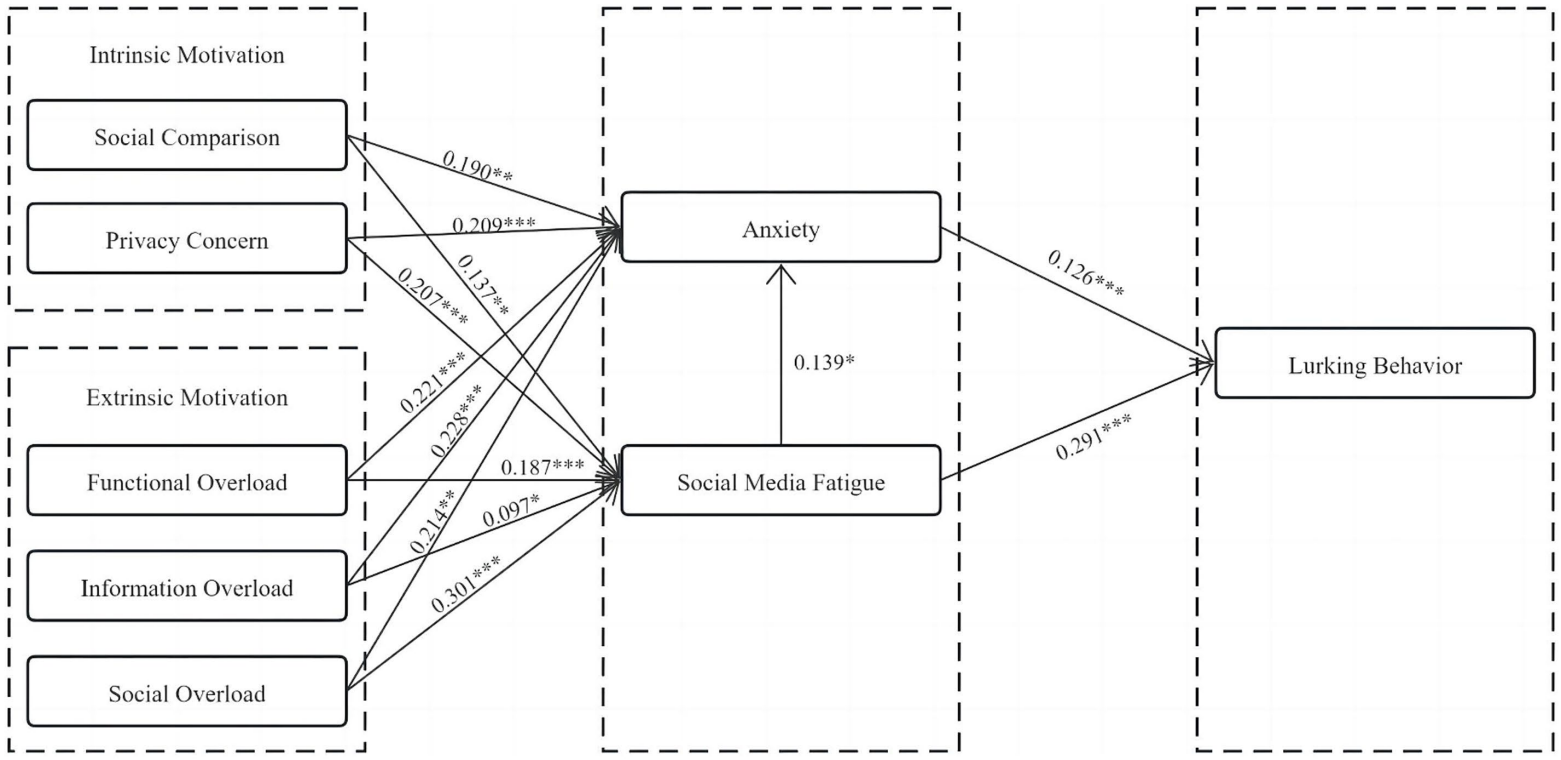
Results of model testing.

Figure 2 illustrates the results of hypothesis testing for structural equation modeling (SEM). In the SSO theoretical framework, stressors stem from both intrinsic and extrinsic motivation dimensions. The results of the data on the intrinsic motivation dimension reveal that social comparison is positively associated with social media fatigue (*β* = 0.137, *p* < 0.01) and anxiety (*β* = 0.190, *p* < 0.01), while privacy concern is positively associated with social media fatigue (*β* = 0.207, *p* < 0.01) and anxiety (*β* = 0.209, *p* < 0.01), supporting hypotheses H1a, H1b, H2a, and H2b.

The findings related to extrinsic motivation show that information overload is positively associated with social media fatigue (*β* = 0.097, *p* < 0.01) and anxiety (*β* = 0.228, *p* < 0.01); functional overload is positively associated with social media fatigue (*β* = 0.187, *p* < 0.01) and anxiety (*β* = 0.221, *p* < 0.01); and social overload is positively associated with social media fatigue (*β* = 0.301, *p* < 0.01) and anxiety (*β* = 0.214, *p* < 0.01), supporting hypotheses H3a, H3b, H4a, H4b, H5a, and H5b.

Regarding the impact of mediating variables on lurking behavior, the data demonstrates that anxiety is positively associated with lurking behavior (*β* = 0.126, *p* < 0.01), supporting hypothesis H6; social media fatigue is positively associated with lurking behavior (*β* = 0.291, *p* < 0.01), supporting hypothesis H7a; additionally, social media fatigue is positively associated with anxiety (*β* = 0.139, *p* < 0.01), supporting hypothesis H7b.

### 5.4 Mediating effects test

To examine the mediation mechanism in our study, we employed the percentile Bootstrap method of bias correction introduced by Hayes (2017) for conducting the mediation effect analysis. With a confidence interval set at 95% and 5,000 iterations of resampling, the results of the analysis indicate that the confidence intervals for all path effects do not contain 0, confirming the presence of mediating effects (Table 5).

**Table 5:** Mediation effect test.

Path	Parameter	Estimate	Proportion of mediated effect in the serial mediation	Lower	Upper	*p*
SC→SMF→AM→LUK	Direct effect	0.151	1.31%	0.082	0.221	0
	Indirect	0.002		0	0.005	0.011
	Total effect	0.153		0.083	0.222	0
PC→SMF→AM→LUK	Direct effect	0.087	2.25%	0.012	0.159	0.023
	Indirect	0.002		0	0.007	0.013
	Total effect	0.089		0.015	0.161	0.018
IO→SMF→AM→LUK	Direct effect	0.176	0.56%	0.098	0.251	0.001
	Indirect	0.001		0	0.004	0.025
	Total effect	0.177		0.1	0.252	0
FO→SMF→AM→LUK	Direct effect	0.197	1%	0.127	0.271	0
	Indirect	0.002		0	0.007	0.011
	Total effect	0.2		0.129	0.272	0
SO→SMF→AM→LUK	Direct effect	0.113	0.26%	0.042	0.183	0.001
	Indirect	0.003		0.001	0.009	0.014
	Total effect	0.117		0.046	0.186	0.001

SC, Social comparison; PC, Privacy concern; FO, Functional overload; IO, Information overload; SO, Social overload; AM, Anxiety; SMF, Social media fatigue; LUK, Lurking behavior.

In the first pathway, social comparison exhibited a significant impact on lurking behavior with a direct effect size of 0.151. Further analysis revealed that social media fatigue and anxiety acted as mediators between social comparison and lurking behavior. Together, social media fatigue and anxiety influenced lurking behavior with an indirect effect size of 0.002, accounting for 1.31%. This suggests that individuals may opt for lurking behavior under the influence of social comparison through the pathways of social media fatigue and anxiety.

Moving on to the second pathway, privacy concern also showed a significant influence on lurking behavior, with a direct effect size of 0.087. Social media fatigue and anxiety played intermediary roles between privacy concern and lurking behavior, collectively affecting lurking behavior with an indirect effect size of 0.002, accounting for 2.25%. This indicates that privacy concerns can trigger social media fatigue and anxiety, subsequently impacting an individual’s lurking behavior.

In the third pathway, information overload displayed a notable impact on lurking behavior with a direct effect size of 0.176. Social media fatigue and anxiety jointly mediated the relationship between information overload and lurking behavior, resulting in an indirect effect size of 0.001, representing 0.56%. This suggests that information overload may lead to social media fatigue and anxiety, thereby influencing users’ decisions to engage in lurking behavior.

Likewise, in the fourth pathway, functional overload significantly influenced lurking behavior with a direct effect size of 0.197. Social media fatigue and anxiety jointly served as mediators between functional overload and lurking behavior, presenting an indirect effect size of 0.002, accounting for 1%. This implies that functional overload could escalate social media fatigue and anxiety, prompting users to seek lurking behavior as a coping mechanism.

The fifth pathway illustrated that social overload had a comparatively minor impact on lurking behavior, with a direct effect size of 0.113. Social media fatigue and anxiety jointly mediated the association between social overload and lurking behavior, resulting in an indirect effect size of 0.003, accounting for 0.26%. This indicates that social overload could instigate social media fatigue and anxiety, subsequently influencing users’ decisions to engage in lurking behavior.

## 6 Discussion

Utilizing the SSO theoretical model as a foundational framework, this study delved into how users’ intrinsic motivational factors and social media’s extrinsic motivational factors of perceptual overload contribute to social media fatigue and anxiety, subsequently prompting lurking behavior. Diverging from prior research that primarily examined lurking behavior through a singular lens (Sun et al., 2014), this study integrates individual internal factors and environmental external factors, incorporating social media fatigue and anxiety as mediating variables to examine their impact on users’ lurking behavior. The findings consistently supported the hypotheses. Intrinsic motives such as social comparison and privacy concerns were found to be positively associated with social media fatigue and anxiety, while extrinsic motives including functional overload, information overload, and social overload were also positively associated with social media fatigue and anxiety. Furthermore, social media fatigue and anxiety were identified as key determinants of users’ lurking behavior. Additionally, by considering the psychological aspects of users, the study confirmed that social media fatigue is positively associated with anxiety.

First, both social comparison and privacy concern, as intrinsic motivational aspects, were positively associated with anxiety and social media fatigue. This aligns with the findings of previous researchers (Ortiz et al., 2018; Nisar et al., 2019; Liu et al., 2020), highlighting the critical role of social comparison and privacy concerns in users’ social media experiences. Social comparison metrics suggest that when users compare themselves to individuals on WeChat they view as more skilled or knowledgeable, it can negatively impact their own self-perceptions. This struggle to manage their self-image within WeChat Moments can heighten anxiety levels and contribute to social media fatigue. The primary reason users refrain from sharing information is the apprehension of personal data breaches and misuse by the mobile app. Users often need to disclose sensitive details like location, phone number, identity information, and home address to access new features or services. Neglecting to address privacy breach risks results in users avoiding specific platform features and services. Hence, the importance of privacy concerns cannot be overstressed, and social media platforms ought to enhance privacy protection technologies to mitigate the exposure of users’ personal data, safeguard information confidentiality, and alleviate privacy anxieties.

Secondly, extrinsic factors such as functional overload, information overload, and social overload have been positively associated with social media fatigue and anxiety. Users are inundated with various stimuli such as group messages, public accounts notifications, new feature introductions, short video recommendations, likes, friends’ comments, advertisements, and other interface elements. When the quantity of these services surpasses the user’s capacity, the user feels the urge to escape from social media. Functional overload, information overload, and social overload collectively play a significant role in contributing to social media fatigue, in accordance with findings by Liu et al. (2020), Lee et al. (2016), and Guo et al. (2020). The saturated social media environment blurs the boundaries between users’ personal and professional lives, causing users to experience pressures related to information processing, technical functions, and social interactions. This results in work-related boundaries encroaching into personal life boundaries, leading to private leisure time being dominated by social media content. The negative user experience stemming from these processes acts as a direct stressor, triggering negative emotions such as disinterest, boredom, and anxiety. In response, users may modify their behaviors by reducing their social media usage, lurking, dropping out, ignoring content, quitting platforms, fleeing from interactions, or transitioning to alternative platforms. This gradual disengagement from social media, as seen in users retiring from WeChat Moments, serves as a defense mechanism to preserve the boundaries between work, social, and personal life domains. This aligns with the findings of Liu et al. (2020) that suggest social media saturation can lead to user anxiety. Active user participation plays a pivotal role in sustaining social media platforms by generating new knowledge and value for business, economic, and public administration purposes. Therefore, social media platforms should prioritize improving their technical infrastructure and services through extensive user research. By refining product design and establishing a secure, streamlined, and user-centric service system, platforms can address user apprehensions, minimize societal repercussions, and address operational hurdles. This strategic approach can enhance users’ emotional satisfaction and foster increased engagement in information dissemination.

Third, the study findings revealed a positive association between social media fatigue and anxiety, with both factors correlating positively with lurking behavior. Prolonged negative emotional states often drive users toward lurking, a passive observation mode devoid of active engagement, as a coping mechanism to alleviate social media-induced stress. Therefore, social media platforms should prioritize addressing users’ negative emotional states by emphasizing the generalization of Moments, optimizing platform management, reducing commercial intrusions in social interactions like WeChat Moments, and offering sophisticated, empathetic tools for managing contextual relationships. These personalized contextual relationship management tools can aid users in navigating intricate social networks, thereby enriching the social ambiance of WeChat Moments interactions and fostering users’ motivation to share information. Additionally, it is recommended that social media platforms introduce features like “short-term lurking” or “short-term disconnection” to empower technology firms and service providers to leverage algorithms for monitoring users’ information consumption, browsing patterns, engagement levels, and screen time. By integrating this data with a meticulous analysis of users’ social media behaviors, these algorithms could automatically suggest users engage in “short-term lurking” or “short-term disconnection” within specified timeframes. This feature should allow user customization and control, enabling individuals to decide whether to enable social media access during these intervals. In the contemporary era of information overload post-connectionism, the notion of “digital escape” is as vital as “digital connectivity” and merits significant consideration.

## 7 Conclusion

### 7.1 Theoretical contributions

Based on the SSO model, our study investigated the lurking behaviors of users on social media platforms and analyzed the effects of social comparison, privacy concerns, information overload, functional overload, and social overload on social media fatigue, anxiety, and lurking behaviors. The contributions of this study will be discussed in the subsequent sections.

With the pervasive influence of social media in the era of big data, the prevalence of negative emotions and detrimental usage patterns among users has emerged as a significant trend. In contrast to prior research primarily concentrating on addictive or persistent usage tendencies, our study investigates the less-explored aspects of prolonged usage, unraveling the mechanisms underlying users’ anxiety and social media fatigue across cognitive, affective, and behavioral dimensions within the social media sphere. Furthermore, it scrutinizes the interplay between users’ lurking behaviors and negative emotions like fatigue and anxiety, bridging a scholarly void that traditionally focused solely on active or continuous usage behaviors, failing to elucidate the intrinsic impact of negative emotions on adverse usage patterns. This unique perspective not only introduces a fresh theoretical framework but also provides pragmatic insights for understanding and addressing these complex dynamics.

Additionally, this study establishes an integrated explanatory model that incorporates intrinsic and extrinsic influencing factors, shifting the discussion from static factor analysis to dynamic mechanism explanation. It provides a comprehensive account of the change mechanism in users’ information behavior from active engagement to lurking. Many previous studies have focused on either extrinsic social factors or intrinsic individual factors when discussing user lurking behaviors (Sun et al., 2014; Ortiz et al., 2018; Nguyen, 2020), neglecting the interplay between these factors and failing to systematically elucidate the mechanism of information behavior change among users. The results suggest that exploring lurking behaviors on social media from both intrinsic motivation and extrinsic motivation perspectives is both reasonable and effective. Perceived high risks associated with privacy maintenance and image management, coupled with the pressure of an overloaded social media environment, influence users’ perceptions of information sharing and online interactions, diminishing the value and necessity of information behavior within their social media platforms. This leads to the emergence of fatigue and anxiety, leading users to transition from active engagement to lurking behaviors.

### 7.2 Practical implications

The study of the factors influencing lurking behavior is essential in the advancement of social media and holds practical significance.

First, social media functions as a dynamic platform enabling content creation and exchange grounded in user relationships. The active engagement of users remains pivotal in driving information dissemination and enriching the vibrancy of social media ecosystems. Yet, lurking behaviors have the potential to obstruct the evolution of social media platforms, impeding operators from efficiently aggregating data, understanding user preferences, and elevating service quality via user-generated content. Our study underscores the significance of users’ inherent psychological stimuli, such as privacy concerns and social comparisons, in diminishing their involvement in online social interactions. Consequently, social media providers must prioritize enhancing and innovating the features and functionalities of their applications to fortify users’ confidence. For instance, empowering users to configure privacy settings for their communications can amplify trust and alleviate concerns about the platform. Furthermore, continual improvements in privacy policy adherence and simplification of application terms and conditions are essential to assure users that their privacy is shielded, thereby bolstering their sense of security.

Second, it is imperative for social media operators to recognize that the benefits derived from different user behaviors vary significantly. Merely increasing the number of registered users without focusing on user activity and engagement is counterproductive. Operators need to carefully address factors that can induce anxiety in users and pay heed to users’ psychological behaviors. Operators can alleviate users’ psychosocial burden by creating a relaxed social environment through technical interventions, as well as implementing measures to automatically detect and block spam to mitigate the stress of perceived social media overload.

Finally, operators should diligently analyze users’ habits and preferences to enhance personalized information recommendations and implement effective information blocking mechanisms. By alleviating users’ tendency to engage in social upward comparison, satisfaction levels and user activity can be significantly improved. By studying the factors that trigger user anxiety and lurking behavior, operators can offer targeted psychological support and interventions to users, thereby encouraging user retention and fostering sustainable development for all stakeholders within the social media business ecosystem.

### 7.3 Limitations and future directions

Our research has several limitations that need to be noted. Firstly, the collected questionnaire samples primarily represent middle-aged and young individuals, with less representation from the elderly group. Additionally, the research area is limited to China, thereby restricting the generalizability of the findings. Future studies should aim to utilize more comprehensive and representative data samples. Secondly, this study focused on lurking phenomenon in WeChat, a popular social media platform in China. However, it is important to note that lurking behavior may vary across different social media platforms such as Tiktok, Facebook, and Twitter. Therefore, it is recommended to extend the study of lurking to other social media platforms to gain a more comprehensive understanding. Lastly, while this study primarily explores the negative outcomes of lurking behavior and provides suggestions for operators to enhance user engagement, it is crucial to acknowledge that there may be rationalities and positive aspects associated with the emergence of lurking. Hence, future research should delve deeper into exploring the positive aspects of social media lurking.

## Data availability statement

The raw data supporting the conclusions of this article will be made available by the authors, without undue reservation.

## Ethics statement

Ethical review and approval was not required for the study on human participants in accordance with the local legislation and institutional requirements. Written informed consent from the patients/ participants or patients/participants legal guardian/next of kin was not required to participate in this study in accordance with the national legislation and the institutional requirements.

## Author contributions

XL: Conceptualization, Data curation, Formal analysis, Investigation, Methodology, Software, Supervision, Writing – original draft, Writing – review & editing. RF: Resources, Supervision, Validation, Writing – review & editing. XC: Methodology, Supervision, Writing – review & editing. YY: Writing – review & editing, Software, Formal analysis, Data curation.

## References

[ref1] Barnidge M. Peacock C. Kim B. Kim Y. Xenos M. A. (2023). Networks and selective avoidance: how social media networks influence unfriending and other avoidance behaviors. Soc. Sci. Comput. Rev. 41, 1017–1038. doi: 10.1177/08944393211069628

[ref2] Bekker H. L. Legare F. Stacey D. O'Connor A. Lemyre L. (2003). Is anxiety a suitable measure of decision aid effectiveness: a systematic review? Patient Educ. Couns. 50, 255–262. doi: 10.1016/S0738-3991(03)00045-4, PMID: 12900095

[ref3] Cain J. A. Imre I. (2022). Everybody wants some: collection and control of personal information, privacy concerns, and social media use. New Media Soc. 24, 2705–2724. doi: 10.1177/14614448211000327

[ref4] Cao X. Sun J. (2018). Exploring the effect of overload on the discontinuous intention of social media users: an S-O-R perspective. Comput. Hum. Behav. 81, 10–18. doi: 10.1016/J.CHB.2017.11.035

[ref5] Chen X. Sun M. X. Wu D. Song X. Y. (2019). Information-sharing behavior on WeChat moments: the role of anonymity, familiarity, and intrinsic motivation. Front. Psychol. 10:2540. doi: 10.3389/FPSYG.2019.02540, PMID: 31798501 PMC6868072

[ref6] Deci E. L. Ryan R. M. (2000). The "what" and "why" of goal pursuits: human needs and the self-determination of behavior. Psychol. Inq. 11, 227–268. doi: 10.1207/S15327965PLI1104_01

[ref7] Deci E. L. Ryan R. M. Vallerand R. J. Pelletier L. G. (1991). Motivation and education: the self-determination perspective. Educ. Psychol. 26, 325–346. doi: 10.1080/00461520.1991.9653137

[ref8] Dhir A. Kaur P. Chen S. Pallesen S. (2019). Antecedents and consequences of social media fatigue. Int. J. Inf. Manag. 48, 193–202. doi: 10.1016/J.IJINFOMGT.2019.05.021

[ref9] Dhir A. Talwar S. Kaur P. Budhiraja S. Islam N. (2021). The dark side of social media: stalking, online self-disclosure and problematic sleep. Int. J. Consum. Stud. 45, 1373–1391. doi: 10.1111/IJCS.12659

[ref10] Dhir A. Yossatorn Y. Kaur P. Chen S. (2018). Online social media fatigue and psychological wellbeing: a study of compulsive use, fear of missing out, fatigue, anxiety and depression. Int. J. Inf. Manag. 40, 141–152. doi: 10.1016/J.IJINFOMGT.2018.01.012

[ref11] Dunbar R. I. M. (1992). Neocortex size as a constraint on group size in primates. J. Hum. Evol. 22, 469–493. doi: 10.1016/0047-2484(92)90081-J

[ref12] Edelmann N. (2013). Reviewing the definitions of "lurkers" and some implications for online research. Cyberpsychol. Behav. Soc. Netw. 16, 645–649. doi: 10.1089/CYBER.2012.0362, PMID: 23848960

[ref13] Fan X. Jiang X. Deng N. Dong X. Lin Y. (2020). Does role conflict influence discontinuous usage intentions? Privacy concerns, social media fatigue and self-esteem. Inf. Technol. People 34, 1152–1174. doi: 10.1108/ITP-08-2019-0416

[ref14] Festinger L. (1954). A theory of social comparison processes. Hum. Relat. 7, 117–140. doi: 10.1177/001872675400700202

[ref15] Fornell C. Larcker D. F. (1981). Evaluating structural equation models with unobservable variables and measurement error. J. Mark. Res. 18, 39–50. doi: 10.1177/002224378101800104

[ref16] Fu S. Li H. Liu Y. Pirkkalainen H. Salo M. (2020). Social media overload, exhaustion, and use discontinuance: examining the effects of information overload, system feature overload, and social overload. Inf. Process. Manag. 57:102307. doi: 10.1016/J.IPM.2020.102307

[ref17] Guo Y. Lu Z. Kuang H. Wang C. (2020). Information avoidance behavior on social network sites: information irrelevance, overload, and the moderating role of time pressure. Int. J. Inf. Manag. 52:102067. doi: 10.1016/J.IJINFOMGT.2020.102067

[ref18] Hayes A. F. (2017). Introduction to mediation, moderation, and conditional process analysis: A regression-based approach. New York: Guilford Publications.

[ref19] Hogan T. P. Brashers D. E. (2015). “The theory of communication and uncertainty management: implications from the wider realm of information behavior” in Uncertainty, information management, and disclosure decisions. eds. Afifi T. Afifi W. (London: Routledge), 45–66.

[ref20] Hong Y. Hu J. Zhao Y. (2023). Would you go invisible on social media? An empirical study on the antecedents of users' lurking behavior. Technol. Forecast. Soc. Change 187:122237. doi: 10.1016/J.TECHFORE.2022.122237

[ref21] Hsu J. S. C. Chiu C. M. Chang-Chien Y. T. Tang K. (2023). How social media fatigue feigning and altering emotion discourage the use of social media. Internet Res. in press 34, 1488–1518. doi: 10.1108/INTR-06-2022-0390

[ref22] Hu B. Zhu Y. Liu C. Zheng S. Zhao Z. Bao R. (2024). Collectivism, face concern and Chinese-style lurking among university students: the moderating role of trait mindfulness. Front. Psychol. 15:1298357. doi: 10.3389/FPSYG.2024.1298357, PMID: 38449746 PMC10915208

[ref23] Huang Q. Lei S. Ni B. (2022). Perceived information overload and unverified information sharing on WeChat amid the COVID-19 pandemic: a moderated mediation model of anxiety and perceived herd. Front. Psychol. 13:837820. doi: 10.3389/FPSYG.2022.837820, PMID: 35185742 PMC8853730

[ref24] Hung S. Y. Durcikova A. Lai H. M. Lin W. M. (2011). The influence of intrinsic and extrinsic motivation on individuals knowledge sharing behavior. Int. J. Hum.-Comput. Stud. 69, 415–427. doi: 10.1016/J.IJHCS.2011.02.004

[ref25] Jabeen F. Tandon A. Sithipolvanichgul J. Srivastava S. Dhir A. (2023). Social media-induced fear of missing out (FoMO) and social media fatigue: the role of narcissism, comparison and disclosure. J. Bus. Res. 159:113693. doi: 10.1016/J.JBUSRES.2023.113693

[ref26] Kang K. Shin S. K. (2008). "A model of virtual community knowledge exchange intentions: Perceived network structure, self-efficacy and individual motivations," in *Proceedings of the 39th decision sciences institute (DSI) annual meeting* (Fresno, TX: Decision Sciences Institute).

[ref27] Karr-Wisniewski P. Lu Y. (2010). When more is too much: operationalizing technology overload and exploring its impact on knowledge worker productivity. Comput. Hum. Behav. 26, 1061–1072. doi: 10.1016/J.CHB.2010.03.008

[ref28] Kaur P. Islam N. Tandon A. Dhir A. (2021). Social media users’ online subjective well-being and fatigue: a network heterogeneity perspective. Technol. Forecast. Soc. Change 172:121039. doi: 10.1016/J.TECHFORE.2021.121039

[ref29] Kim J. Kim H. M. Kim M. (2021). The impact of a sense of virtual community on online community: does online privacy concern matter? Internet Res. 31, 519–539. doi: 10.1108/INTR-01-2020-0015

[ref30] Kim S. Park H. (2015). Empirical study on antecedents and consequences of users' fatigue on SNS and the moderating effect of habit. J. Korea Soc. IT Serv. 14, 137–157. doi: 10.9716/KITS.2015.14.4.137

[ref31] Koeske G. F. Koeske R. D. (1993). A preliminary test of a stress-strain-outcome model for reconceptualizing the burnout phenomenon. J. Soc. Serv. Res. 17, 107–135. doi: 10.1300/J079V17N03_06

[ref32] Lee A. R. Son S. M. Kim K. K. (2016). Information and communication technology overload and social networking service fatigue: a stress perspective. Comput. Hum. Behav. 55, 51–61. doi: 10.1016/J.CHB.2015.08.011

[ref33] Li J. Guo F. Qu Q. X. Hao D. (2022). How does perceived overload in mobile social media influence users’ passive usage intentions? Considering the mediating roles of privacy concerns and social media fatigue. Int. J. Hum. Comput. Interact. 38, 983–992. doi: 10.1080/10447318.2021.1986318

[ref34] Li K. Jiang S. Yan X. Li J. (2024). Mechanism study of social media overload on health self-efficacy and anxiety. Heliyon 10:E23326. doi: 10.1016/J.HELIYON.2023.E23326, PMID: 38163164 PMC10757012

[ref35] Lim M. Yang Y. (2019). Upward social comparison and Facebook users’ grandiosity: examining the effect of envy on loneliness and subjective well-being. Online Inf. Rev. 43, 635–652. doi: 10.1108/OIR-04-2017-0137

[ref36] Liu X. Min Q. Wu D. Liu Z. (2020). How does social network diversity affect users’ lurking intention toward social network services? A role perspective. Inf. Manag. 57:103258. doi: 10.1016/J.IM.2019.103258

[ref37] Luo L. (1999). Work motivation, job stress and employees' well-being. J. Appl. Manag. Stud. 8, 61–72.

[ref38] Maier C. Laumer S. Eckhardt A. Weitzel T. (2015). Giving too much social support: social overload on social networking sites. Eur. J. Inf. Syst. 24, 447–464. doi: 10.1057/EJIS.2014.3

[ref39] Neelen M. Fetter S. (2010). Lurking: a challenge or a fruitful strategy? A comparison between lurkers and active participants in an online corporate community of practice. Int. J. Knowl. Learn. 6, 269–284. doi: 10.1504/IJKL.2010.038649

[ref40] Nguyen T. M. (2020). Four-dimensional model: a literature review on reasons behind lurking behavior. VINE J. Inf. Knowl. Manag. Syst. 51, 302–317. doi: 10.1108/VJIKMS-10-2019-0168

[ref41] Nguyen T. M. (2021). Four-dimensional model: a literature review in online organisational knowledge sharing. VINE J. Inf. Knowl. Manag. Syst. 51, 109–138. doi: 10.1108/VJIKMS-05-2019-0077

[ref42] Nguyen T. M. Viet Ngo L. Paramita W. (2022). Turning lurkers into innovation agents: an interactionist perspective of self-determinant theory. J. Bus. Res. 141, 822–835. doi: 10.1016/J.JBUSRES.2021.11.087

[ref43] Nisar T. M. Prabhakar G. Ilavarasan P. V. Baabdullah A. M. (2019). Facebook usage and mental health: an empirical study of role of non-directional social comparisons in the UK. Int. J. Inf. Manag. 48, 53–62. doi: 10.1016/J.IJINFOMGT.2019.01.017

[ref44] Okano H. Nomura M. (2023). Examining social anxiety and dual aspects of social comparison orientation: the moderating role of self-evaluation of social skills. Front. Psychol. 14:1270143. doi: 10.3389/FPSYG.2023.1270143, PMID: 38144985 PMC10748495

[ref45] Olatokun W. Nwafor C. I. (2012). The effect of extrinsic and intrinsic motivation on knowledge sharing intentions of civil servants in Ebonyi state, Nigeria. Inf. Dev. 28, 216–234. doi: 10.1177/0266666912438567

[ref46] Ortiz J. Chih W. H. Tsai F. S. (2018). Information privacy, consumer alienation, and lurking behavior in social networking sites. Comput. Hum. Behav. 80, 143–157. doi: 10.1016/J.CHB.2017.11.005

[ref47] Osatuyi B. (2015). Is lurking an anxiety-masking strategy on social media sites? The effects of lurking and computer anxiety on explaining information privacy concern on social media platforms. Comput. Hum. Behav. 49, 324–332. doi: 10.1016/J.CHB.2015.02.062

[ref48] Reer F. Tang W. Y. Quandt T. (2019). Psychosocial well-being and social media engagement: the mediating roles of social comparison orientation and fear of missing out. New Media Soc. 21, 1486–1505. doi: 10.1177/1461444818823719

[ref49] Sun N. Rau P. P. L. Ma L. (2014). Understanding lurkers in online communities: a literature review. Comput. Hum. Behav. 38, 110–117. doi: 10.1016/J.CHB.2014.05.022

[ref50] Takahashi M. Fujimoto M. Yamasaki N. (2003). "The active lurker: influence of an in-house online community on its outside environment," in *Proceedings of the 2003 ACM international conference on supporting group work* (New York: Association for Computing Machinery), 1–10.

[ref51] Teo T. S. H. Lim V. K. G. Lai R. Y. C. (1999). Intrinsic and extrinsic motivation in internet usage. Omega 27, 25–37. doi: 10.1016/S0305-0483(98)00028-0

[ref52] Thompson D. V. Hamilton R. W. Rust R. T. (2005). Feature fatigue: when product capabilities become too much of a good thing. J. Mark. Res. 42, 431–442. doi: 10.1509/JMKR.2005.42.4.431

[ref53] Vallerand R. (2001). “JA hierarchical model of intrinsic and extrinsic motivation in sport and exercise” in Advances in motivation in sport and exercise. eds. Roberts G. C. Treasure D. (Champaign, IL: Human Kinetics), 263–319.

[ref54] Van Mierlo T. (2014). The 1% rule in four digital health social networks: an observational study. J. Med. Internet Res. 16:e2966. doi: 10.2196/JMIR.2966, PMID: 24496109 PMC3939180

[ref55] Venkatesh V. Speier C. Morris M. G. (2002). User acceptance enablers in individual decision making about technology: towards an integrated model. Decis. Sci. 33, 297–316. doi: 10.1111/J.1540-5915.2002.TB01646.X

[ref56] Verduyn P. Gugushvili N. Massar K. Täht K. Kross E. (2020). Social comparison on social networking sites. Curr. Opin. Psychol. 36, 32–37. doi: 10.1016/J.COPSYC.2020.04.002 32387840

[ref57] Winder D. (2021). Unsecured Facebook databases leak data of 419 million users. Available at: https://www.forbes.com/sites/daveywinder/2019/09/05/facebook-security-snafu-exposes-419-million-user-phone-numbers/?sh526d8da141ab7 [Accessed May 1, 2024].

[ref58] Zhang Y. He W. Peng L. (2022). How perceived pressure affects users’ social media fatigue behavior: a case on WeChat. J. Comput. Inf. Syst. 62, 337–348. doi: 10.1080/08874417.2020.1824596

[ref59] Zhang Y. Shi S. Guo S. Chen X. Piao Z. (2021). Audience management, online turbulence and lurking in social networking services: a transactional process of stress perspective. Int. J. Inf. Manag. 56:102233. doi: 10.1016/J.IJINFOMGT.2020.102233

[ref60] Zhang S. Zhao L. Lu Y. Yang J. (2016). Do you get tired of socializing? An empirical explanation of discontinuous usage behaviour in social network services. Inf. Manag. 53, 904–914. doi: 10.1016/J.IM.2016.03.006

[ref61] Zhou T. Xie Y. (2023). Understanding social media users' information avoidance intention: a C-A-C perspective. Aslib J. Inf. Manag. 76, 570–584. doi: 10.1108/AJIM-10-2022-0471

