# Supplementary material for: “Left on read” examining social media users’ lurking behavior: an integration of anxiety and social media fatigue
Source: Front Psychol. 2024 Aug 2;15:1406895. doi: 10.3389/fpsyg.2024.1406895 (PMC11327116; doi:10.3389/fpsyg.2024.1406895)
Supplement: Supplementary file 1 [file Table_1.DOCX]

Supplementary Material

**Supplementary Table 1.** Measurements of key variables.

| Constructs |  | Used measurement items | Resource |
| --- | --- | --- | --- |
| Social  Comparison | SC1 | On WeChat, I often compare my achievements in life with others. | Nisar et al. (2019); Jabeen et al. (2023) |
|  | SC2 | On WeChat, I am less confident about what I have achieved compared to other people. |  |
|  | SC3 | On WeChat, I sometimes compare myself to people who have achieved more than me. |  |
|  | SC4 | On WeChat, I pay a lot of attention to how I do things compared to others. |  |
|  | SC5 | I often consult others before posting on WeChat Moments. |  |
|  | SC6 | I delete posts that don't get a certain number of likes or comments. |  |
|  | SC7 | I feel that I am not as happy as others when I browse my friends' posts. |  |
| Privacy  Concern | PC1 | l am concerned that WeChat would sell my stored personal information in their databases to other companies. | Cain and Imre (2022) |
|  | PC2 | I am concerned that WeChat would share my stored personal information in their databases with other companies without my authorization. |  |
|  | PC3 | I am concerned that WeChat would use my stored personal information for their own advantage or profit. |  |
|  | PC4 | I am concerned that WeChat are collecting personal information about me. |  |
|  | PC5 | It usually bothers me when I do not have control of personal information that provide to WeChat. |  |
|  | PC6 | When WeChat ask me for personal information, I sometimes think twice before providing it. |  |
| Functional  Overload | FO1 | I am often distracted by system functions included on WeChat that are not necessary. | Lee et al. (2016); Guo et al. (2020) |
|  | FO2 | Many functions on WeChat are not relevant to my primary purpose of use. |  |
|  | FO3 | Many of the new functions on WeChat are useless to me. |  |
|  | FO4 | Some functions on WeChat are too complicated for me. |  |
|  | FO5 | Many functions on WeChat tend to try to be too helpful which makes performing my task even harder. |  |
| Information  Overload | IO1 | I am distracted by too much information on WeChat. | Zhang et al. (2016); Guo et al. (2020) |
|  | IO2 | There are so many messages to check and respond on WeChat that I often feel overwhelmed. |  |
|  | IO3 | I find it hard to extract important information from the excessive amount of information available to me on WeChat. |  |
|  | IO4 | I find it hard to get the information that is relevant to my needs from the excessive amount of information available to me on WeChat. |  |
|  | IO5 | I feel that there is a lot of information in WeChat that needs to be consolidated. |  |
| Social  Overload | SO1 | Not all of my WeChat friends are familiar people. | Fu et al. (2020); Guo et al. (2020) |
|  | SO2 | Some WeChat groups have a lot of group messages even though many people don't know each other. |  |
|  | SO3 | I spend too much energy and time replying to my friends' messages on WeChat. |  |
|  | SO4 | There are often friends on WeChat who need me to like, retweet and share, vote, etc. |  |
| Anxiety | AM1 | On WeChat, I get nervous if I have to interact with someone in authority (teacher, boss, etc.). | Liu et al. (2020) |
|  | AM2 | On WeChat, I worry about expressing myself in case I appear awkward. |  |
|  | AM3 | On WeChat, I worry that the information I post will be ignored. |  |
|  | AM4 | On WeChat, I worry about saying something embarrassing. |  |
| Social Media  Fatigue | SMF1 | Sometimes I get bored when using WeChat Moments. | Kaur et al. (2021); Jabeen et al. (2023) |
|  | SMF2 | I find that sometimes I don't feel relaxed even after using WeChat. |  |
|  | SMF3 | After using WeChat for a while, I feel fatigued and it's more difficult to devote myself to study and work. |  |
|  | SMF4 | I sometimes feel that using WeChat has become a task rather than entertainment. |  |
|  | SMF5 | I feel disinterested in whether there are new things happening on WeChat Moments. |  |
|  | SMF6 | I feel indifferent about the reminders or alerts about new things on WeChat Moments. |  |
| Lurking  Behavior | LUK1 | I rarely post updates on WeChat Moments. | Zhang et al. (2021); Hong et al. (2023) |
|  | LUK2 | I rarely like others' posts on WeChat Moments. |  |
|  | LUK3 | I seldom comment on others' posts on WeChat Moments. |  |
|  | LUK4 | I often browse videos or pictures on WeChat Moments, but rarely share them with others. |  |
|  | LUK5 | I often read tweets and comments on WeChat Moments, but rarely interact with others. |  |
